# Assessment of long-term outcomes following Milligan-Morgan hemorrhoidectomy with Doppler transperineal ultrasound and endoscopy: a pilot study

**DOI:** 10.1007/s00384-025-04894-x

**Published:** 2025-05-01

**Authors:** Gianpiero Gravante, Veronica De Simone, Roberto Sorge, Marco La Torre, Vito D’Andrea, Stefania Romano, Gaetano Gallo

**Affiliations:** 1 Department of General Surgery, Azienda Sanitaria Locale ASL Lecce, Casarano, Italy; 2 Proctology and Pelvic Floor Surgery Unit, Ospedale Isola Tiberina-Gemelli Isola, 00186 Rome, Italy; 3 Department of Human Physiology, Laboratory of Biometry, University of Tor Vergata in Rome, Rome, Italy; 4 Department of Surgery, Ospedale Cristo Re, Rome, Italy; 5 Department of Surgery, “Sapienza” University of Rome, Rome, Italy

**Keywords:** Ultrasound, Pelvic floor diseases, Proctology, Hemorrhoids, Hemorrhoidectomy

## Abstract

**Purpose:**

Hemorrhoidectomy remains the gold-standard treatment for advanced hemorrhoidal disease, but long-term outcomes vary depending on the surgical technique and assessment method. This study aims to show the long-term results achieved using a combination of transperineal ultrasound (TPUS) and endoscopy in patients who underwent Milligan-Morgan hemorrhoidectomy (MM).

**Methods:**

A consecutive series of MM patients treated between February 2020 and December 2023 were contacted and underwent a clinical proctological evaluation, a Doppler TPUS, and an endoscopic check of the anal canal. All investigations were performed using the ESAOTE MyLab XPRO80. TPUS anatomy, including Doppler views, was assessed on the axial, sagittal, and coronal planes. Outcome measures consisted of the description of Doppler TPUS modifications following MM, and their relationship with clinical and endoscopic findings.

**Results:**

Twenty-three patients were available for the analysis at 26 months of follow-up (range 14–48). Fifteen patients had a Doppler vascular pattern (65.2%), eight patients a scattered pattern (34.8%). No correlation was present between residual symptoms and the Doppler vascular pattern (*p* = 0.089). The vascular pattern was associated with endoscopic signs of recurrences (*p* = 0.003), and its absence was able to predict the lack of internal recurrences in all patients (100%).

**Conclusion:**

Doppler TPUS could help in the long-term assessment of patients presenting residual symptoms after MM. If confirmed in larger future cohort studies, the 100% negative predictive value of the vascular pattern could be used to exclude internal recurrences during postoperative follow-up.

## Introduction

The transperineal ultrasound (TPUS) is one of the sonographic options for the assessment of pelvic floor diseases, along with both the endoanal and the transvaginal approaches [1, 2]. Although endoanal and transvaginal probes are necessary for the definition of pelvic floor muscles and fascias, and represent the gold-standard techniques for the assessment of obstetric injuries and perianal sepsis, they are not readily available in non-referral tertiary hospitals and may alter the mucosal anatomy and vascularization by exerting a direct pressure on tissues, thereby changing the US signals derived.

TPUS would be more “physiologic” for the study of proctologic diseases, as no internal pressure is applied [3]. Convex and linear probes has already been used on idiopathic perianal sepsis [4–6], perianal Crohn’s [7–9] and anal incontinence [3, 10]. Hemorrhoidal disease (HD), a widespread vascular disease, has generally been left apart. In 2010, Zbar et al. measured the anal cushions area in a group of normal subjects (*n* = 22), in patients affected by III and IV degree HD (*n* = 36) and in 31 who underwent hemorrhoidectomy [11]. Probe wavelength included a 7.5–10 MHz curvilinear transducer (C4 - 7 and C8 - 12) and a 5–10 MHz linear transducer. Results found that TPUS is easy to perform, reproducible, and shows marked differences between normal individuals, patients with symptomatic HD, and patients after hemorrhoidectomy [11]. Limitations consisted of the small sample size of the study, which impairs the generalizability of the findings, and the lack of comparison with other well-established diagnostic modalities of HD, like anoscopy or flexible sigmoidoscopy.

Despite the widespread use of TPUS in other proctologic and pelvic floor disorders, its role in assessing long-term outcomes of hemorrhoidectomy remains poorly explored. The current study aims to show long-term results achieved in our hospital on patients with III–IV degree HD that underwent Milligan-Morgan hemorrhoidectomy (MM), assessed with a clinical proctological evaluation, a Doppler TPUS, and an endoscopic check of the anorectum.

## Materials and methods

This is a prospective observational study and has been reported according to the Strengthening the Reporting of Observational Studies in Epidemiology (STROBE) guideline [12]. All procedures in studies involving human participants were performed by the ethical standards of the institutional and/or national research committee and with the 1964 Helsinki Declaration and its later amendments or comparable ethical standards. The study was approved by the Local Ethical Committee (IRCSS Istituto Oncologico “Gabriella Serio” Prot. 2146/CEL).

Included patients were those with III–IV-degree HD treated with MM between February 2020 and December 2023 in a district general hospital (Azienda Sanitaria Locale ASL Lecce, “Francesco Ferrari” Hospital, Casarano). Excluded patients were those with associated anorectal diseases (i.e., anal fistulas or abscesses, anal fissures, condylomas, tumors, rectocele, internal intussusception, rectal prolapse, ulcerative colitis, Crohn’s disease or any other anoperineal disease), that received previous anorectal surgery or hemorrhoidal procedures other than MM, and with less than one year of follow-up from MM. All operations, as well as clinical, TPUS, and endoscopic assessments, were performed by a well-trained colorectal surgeon (GGr) with more than 20 years of activity, 700 proctologic operations, and 5000 endoscopic procedures. TPUS images were further reviewed by a second Author (GGa), and discrepancies were resolved by discussion or consultation with a third author (VDS).

### Routine perioperative management

Patients were assessed preoperatively with history and a proctological evaluation, including a perineal and digital rectal examination, and anoscopy. When bleeding per rectum or other symptoms suspicious for colorectal cancer were present, a complete colonoscopy or computed tomography colonography was performed before surgery (unless a recent one was already available). Indications for MM consisted of symptomatic III–IV-degree HD according to the Goligher classification [13, 14]. Following surgery, all patients were routinely followed up after 1, 3, and 6 weeks in our outpatient clinic. On these occasions they were asked about the presence of bleeding or pain, and a digital rectal examination was performed to assess the calibre of the anal canal and the tone of the sphincters (both resting and squeeze pressures).

### Study protocol

Between November 2024 and February 2025, patients were selected from a prospectively maintained database according to the inclusion and exclusion criteria, were contacted by phone, and the study protocol was explained. Subsequently, those who agreed underwent a single-stop outpatient clinic visit consisting of an interview about pre- and postoperative residual symptoms and a complete proctological evaluation that included TPUS and endoscopy. Data collected from the original database consisted of basic demographics (age, sex), American Society of Anesthesiologists (ASA) score, and date of surgery.

During the clinic, patients were asked about the presence of residual hemorrhoidal symptoms. Furthermore, a modified version of the Patient Reported Outcome Measure‐Hemorrhoidal Impact and Satisfaction Score (PROM‐HISS) was calculated and recorded for both the preoperative and the current follow-up visit [15]. The PROM-HISS score consists of three domains: five HD symptoms (bleeding, pain, prolapse, soiling, itching; each is scored from 1 — less worrying to 5 — most worrying), impact of symptoms on daily activities (score 0 — lowest to 10 — highest impact), and satisfaction with treatment (score 0 — lowest to 10 — highest satisfaction) [15]. In order to be able to compare the pre- and postoperative values of the score, the third domain, satisfaction with the treatment received, was not included because not available before surgery.

### Radiology

All investigations were performed using the ESAOTE MyLab XPRO80® (Genova-Italy). Five probes were available: C1 - 8 Convex Array transducer — frequency range 1.0–8.0 MHz; L4 - 15 Linear Array transducer — 4.0–15.0 MHz; mC3 - 11 microConvex Array Transducer — 3.0–11.0 MHz; L8 - 24 Linear Array transducer — 8.0–24.0 MHz; L3 - 11 Linear Array Transducer — 3.0–11.0 MHz. Based on our preliminary experience, the most adapt for the assessment of proctological diseases is the mC3 - 11 microConvex Array Transducer: its spatial resolution is adequate for approximately 8-cm depth and the peculiar shape allows easily to scan all axes (sagittal, coronal, axial); linear probes are incapacitated on the coronal and axial views due to the pelvic bony prominences that limit movements and angulations.

**Fig. 1:**
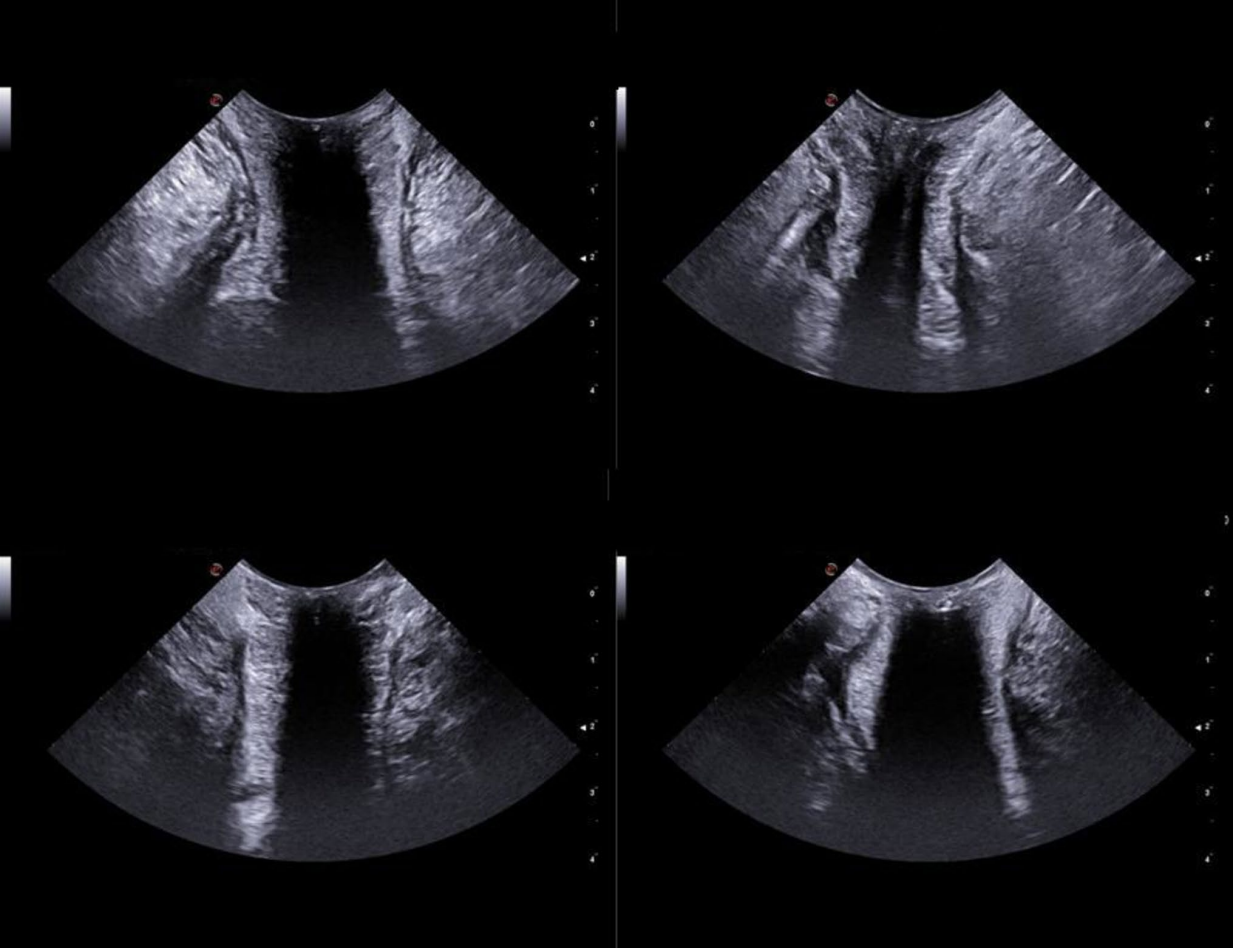
Images achieved with the mC3 - 11 probe (sagittal and coronal view)

**Fig. 2:**
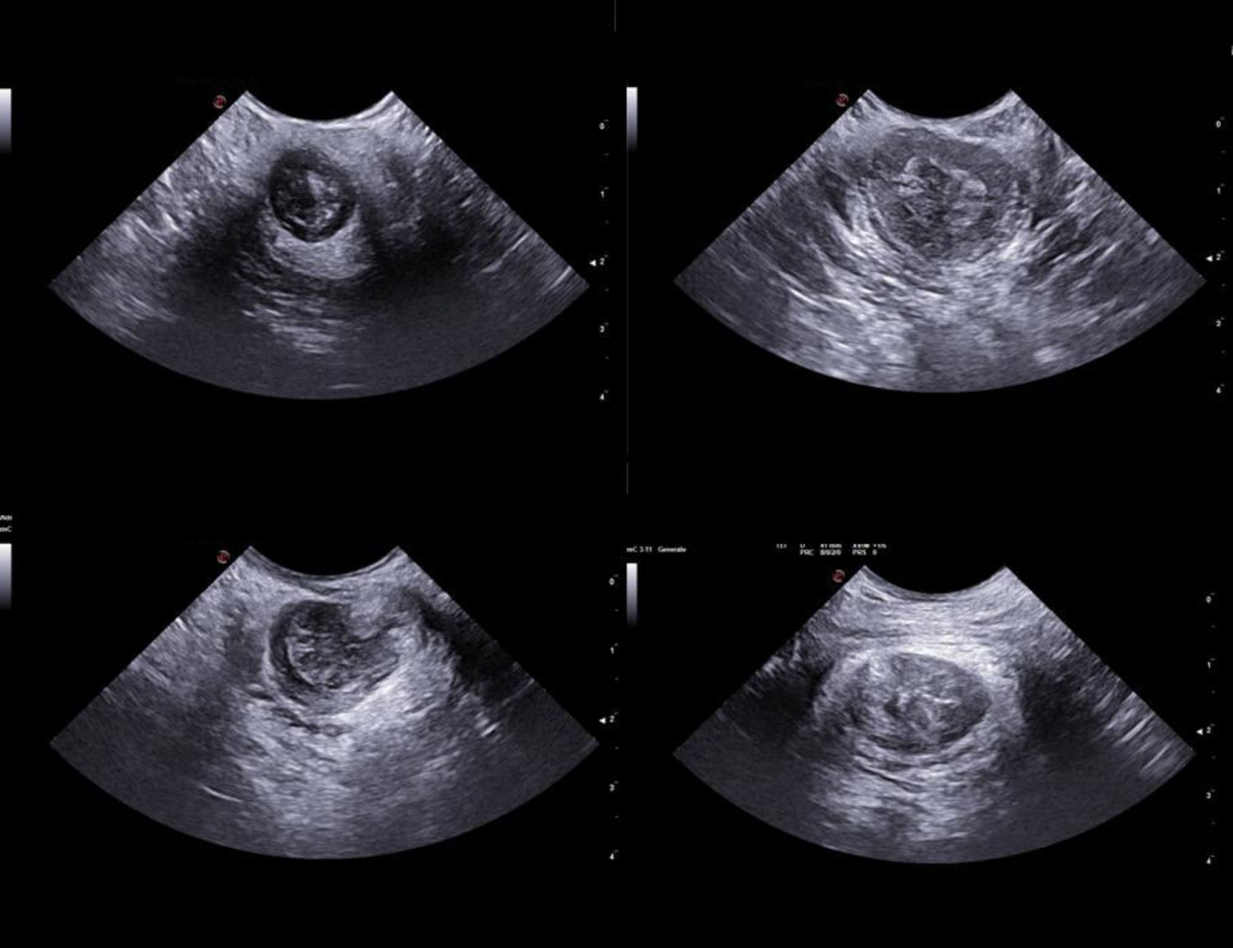
Images achieved with the mC3 - 11 probe (axial view)

**Fig. 3:**
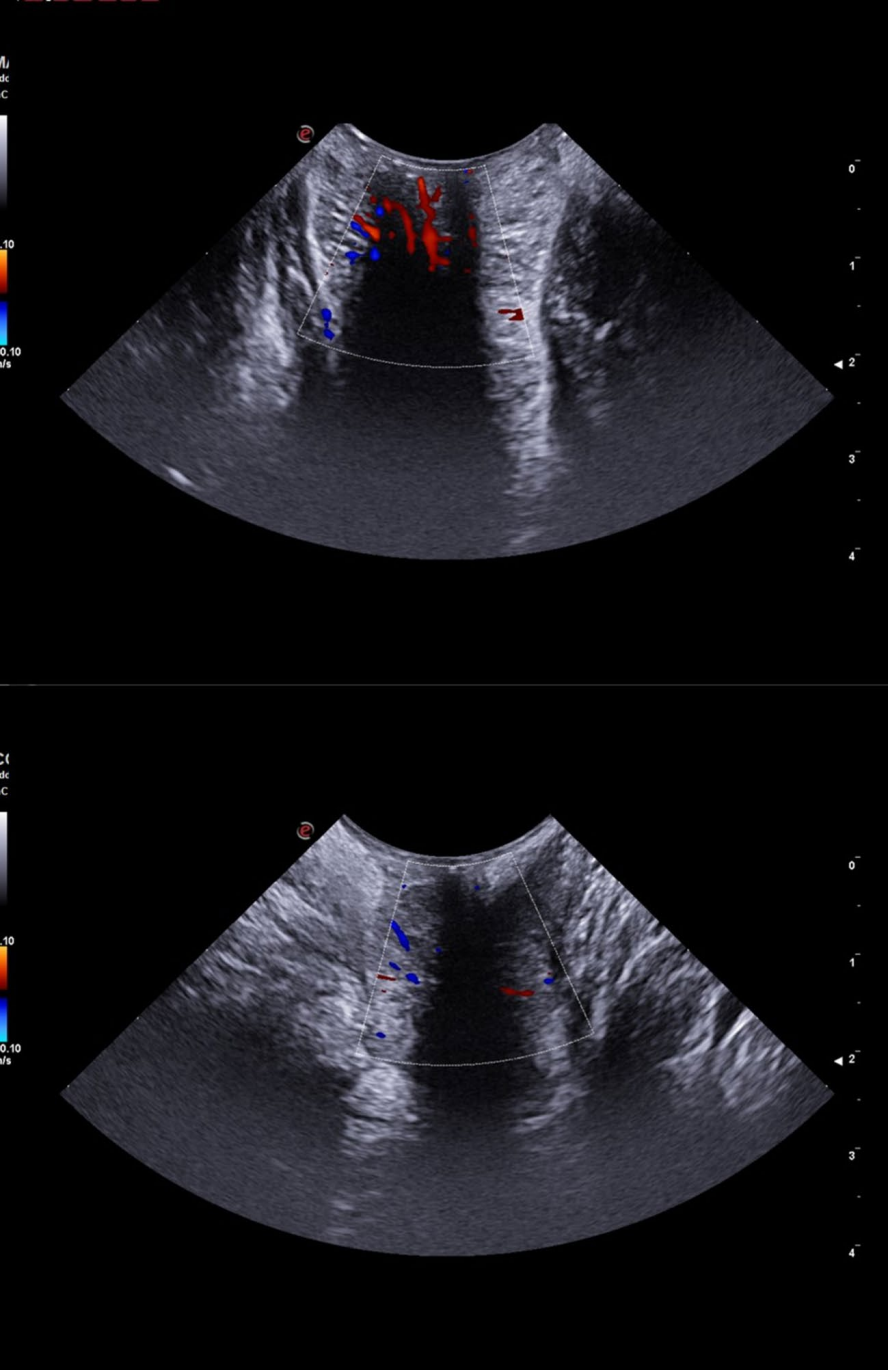
Patterns of Doppler vascularity: vascular (*top panel*) and scattered (*bottom panel*)

All scans have been performed via the perineal approach with the mC3 - 11 probe gently touching the anal verge. For each patient, three Doppler images, one for each anatomic axis (sagittal, coronal, and axial), were collected at rest with no Valsalva manoeuvre (Fig. 1–2). A minimal pressure was exerted on the anal verge to achieve a complete view of the anal canal mucosa without closing the internal vessels. The structural anatomy has been interpreted according to previous reports [16, 17]. Two patterns of vascularity were assigned (Fig. 3). The first pattern, “vascular”, identified a complex vascular architecture consisting of multiple tortuous submucosal vessels that display a robust and widespread arterial signal. Dynamically, the vascular flow was characterized by high-velocity, pulsatile arterial signals in synchrony with the cardiac cycle, prominent systolic pulsations, and a lower, continuous, diastolic component. The second pattern, “scattered”, corresponded to smaller calibre arteries (small coloured dots), less prominent pulsations, and less differences between systole and diastole (Fig. 3). TPUS images were recorded with a non-recognizable numeric code corresponding to the patient’s identity on the main database.

### Endoscopy

Endoscopic images of the anorectum have been collected with the Pentax EPK i7010 column and the E38-i10L colonoscope. For each patient, four images were gathered by rotating the scope every 90° during the retroflexion manoeuvre. Images were stored anonymously using the same corresponding numeric code used for the TPUS. On subsequent analysis, “no recurrence” or “recurrence” was interpreted according to the presence or absence of internal vascular congestion.

### Outcomes

The primary outcome of this study was to describe the radiological anatomy of the anal canal using the TPUS along the three axes. Secondary outcomes were to define which probes, among those available, were most sensitive for the definition of the anal canal anatomy, examine postoperative alterations, and assess the eventual utility and results achieved with the Doppler assessment. US findings were checked with the retroflexed endoscopic views as gold standard for eventual internal recurrences.

### Statistical analysis

All data were inserted into an Excel database (Microsoft, Redmond, Washington, USA) and analyzed with the Statistical Package for the Social Sciences Windows version 27.0 (SPSS, Chicago, Illinois, USA). Descriptive statistics used were the mean ± standard deviation for continuous parametric variables, the median and range for continuous non-parametric variables, and frequencies for categorical variables. Normality assumptions were demonstrated with histograms and the Shapiro–Wilk test. Analysis of comparison between groups was conducted with the ANOVA one-way test for continuous parametric variables, Wilcoxon test for continuous non-parametric variables, and Chi-Square test for categorical variables (Fisher’s exact test if the counts in cells were inferior to 5). A *p* value less than 0.05 was considered statistically significant.

## Results

**Fig. 4:**
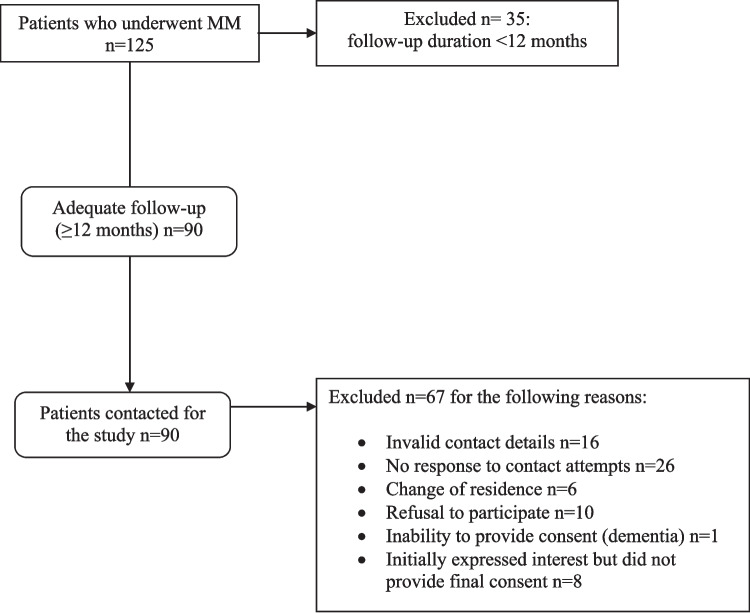
Flow chart illustrating the selection and enrolment of patients in the study. MM: Milligan-Morgan hemorrhoidectomy

The selection process of patients included in the study is illustrated in Fig. 4: ninety out of 125 patients who underwent MM met the inclusion criteria, but ultimately only 23 agreed and were enrolled in the study. Descriptive statistics are presented in Table 1. Most patients were males and all had an ASA score I or II (Table 1). A significant difference was present between the preoperative and postoperative HISS score (Wilcoxon test *p* < 0.001). Eleven patients (47.8%) had no residual symptoms at the time of follow-up, the remaining twelve still claimed a significant improvement after MM. In particular, five patients referred spots of occasional bleeding, two soiling, two pain on defecation, two itching and one a residual prolapse. On physical exam, sixteen patients (69.6%) had no recurrent hemorrhoidal piles or skin tags.

**Table 1:** Descriptive statistics of the cohort of patients

Age (years)	58 ± 12
Sex (males; %)	18 (78.3%)
ASA score	
• I	4 (17.4%)
• II	19 (82.6%)
Follow-up (months)	26 (14–48)
HISS score	
• Preoperative	25 (range 13–35)
• Postoperative	6 (range 5–24)
Residual symptoms (*n*; %)	11 (47.8%)

### Radiology

**Fig. 5:**
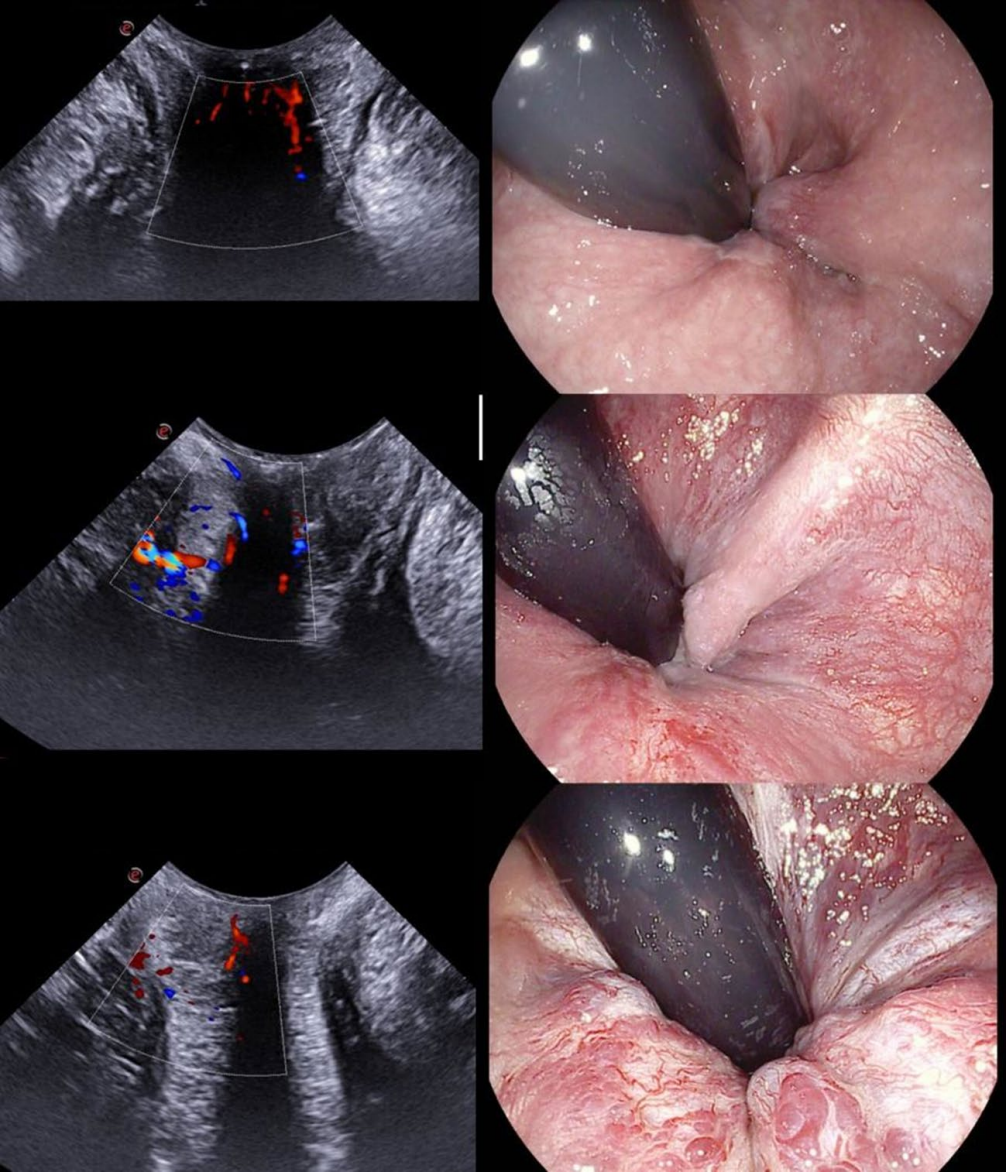
Comparison of the vascular pattern on transperineal US Doppler (*left panels*) with corresponding endoscopic images (*right panels*)

**Fig. 6:**
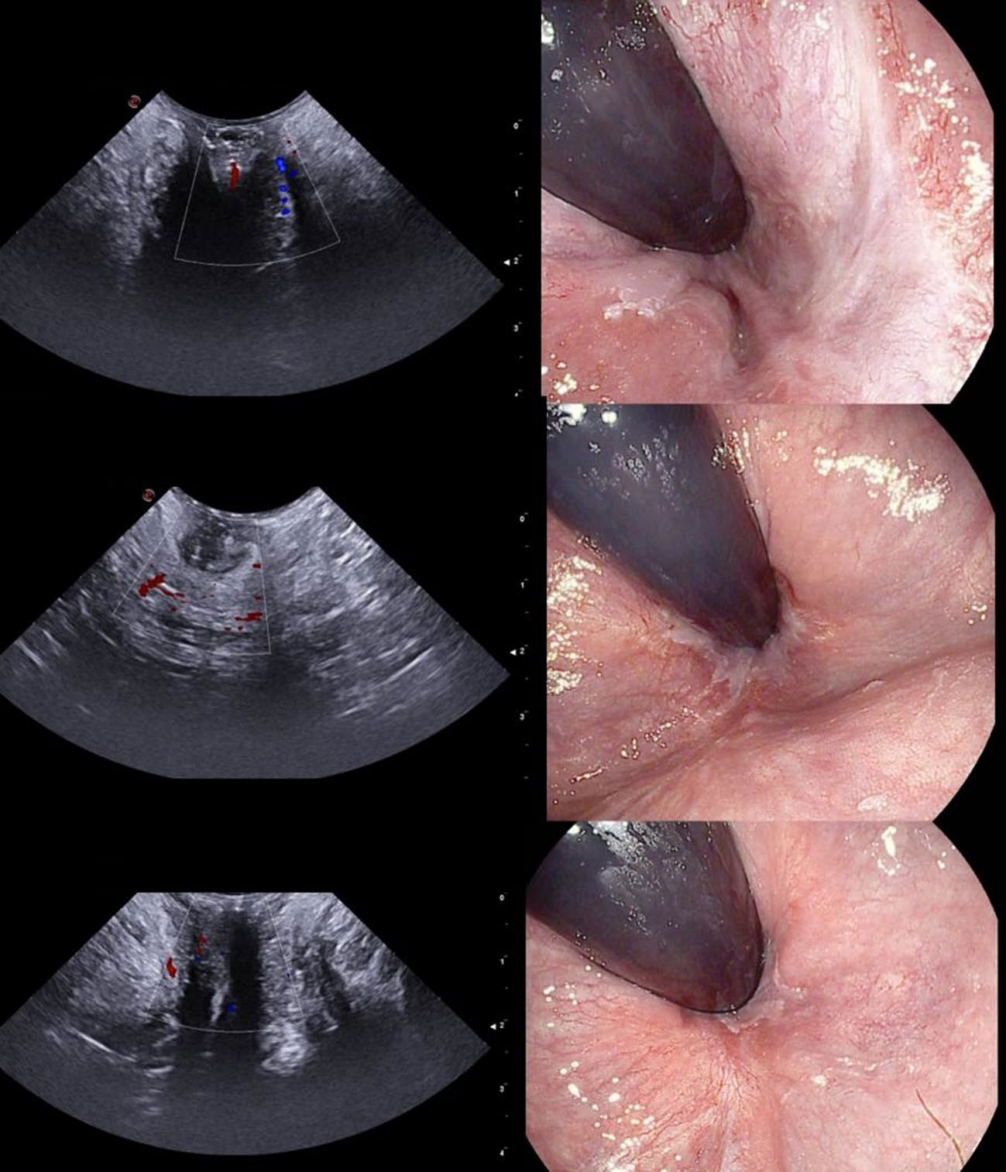
Comparison of the scattered pattern on transperineal US Doppler (left panels) with corresponding endoscopic images (*right panels*)

The Doppler study uncovered the local tissue vascularization. Fifteen patients had a vascular pattern (65.2%; Fig. 5) and eight patients a scattered pattern (34.8%; Fig. 6). There was no association between the presence of residual symptoms and the type of Doppler US pattern (Fisher’s exact test 2-sided, *p* = 0.089).

### Endoscopic correlation

The endoscopic analysis showed that ten patients had endoscopic signs of recurrence (43.5%; Fig. 5–6). A significant correlation was present between the presence of residual symptoms and the endoscopic findings (Fisher’s exact test 2-sided, *p* = 0.036). Endoscopic recurrences were also significantly associated with the Doppler US vascular pattern (Fisher’s exact test, 2-sided, *p* = 0.003). The positive predictive value of the US vascular pattern was 66.7% (95% CI: 50.1%–79.9%) and the negative predictive value was 100% (95% CI: 63.1%–100%) (Table 2).

**Table 2:** Results of TPUS as a diagnostic test for hemorrhoidal disease recurrence

Statistic	Value	95% CI
Sensitivity	100%	69.1% to 100%
Specificity	61.5%	31.6% to 86.1%
Positive Likelihood Ratio	2.6	1.3 to 5.2
Negative Likelihood Ratio	0	
Disease prevalence	43.5%	23.2% to 65.5%
Positive Predictive Value	66.7%	50.1% to 79.9%
Negative Predictive Value	100%	63.1% to 100%
Accuracy	78.3%	56.3% to 92.5%

## Discussions

The present study had two major purposes. The first originated from the scarcity of data describing the US normal anatomy of the anal region with the transperineal approach. Images of the anorectal normal anatomy were already available only for the axial plane [10, 16–19]. All these studies focused on the application of TPUS to investigate obstetric anal sphincter injuries (OASIS) and started with the description of the sonographic appearance of the normal anal sphincter complex. The initial purpose of the present study was to describe US findings in other planes (sagittal and coronal) still uninvestigated with the transperineal approach. Based on our personal experience, the probe with a tapered shape that created relatively higher wavelengths was the most useful for the purpose (microConvex 3–11). The anatomy described in this study showed similar sonographic appearances of the sphincters complex to those already published (hypoechoic the internal one, hyperechoic the external one). In the axial plane our images presented the internal anal sphincter as an hypoechoic ring, the anal mucosa as mixed echogenicity bunched image, and the external sphincter as a hyperechoic less-defined ring (Fig. 2) — similarly to Huang and colleagues [16]. The absence of internal compression allowed a clearer and better-defined appearance of the anal mucosa as a thick layer of mixed echogenicity. This was more visible in the sagittal and coronal images when aligning the US waves with the anal canal. In this setting, the thick mixed-echoic layers corresponded to the anal canal mucosa, located anteriorly and posteriorly to a hypoechoic central region that corresponded to the air inside the anal canal. The internal sphincter was as thin hypoechoic streaks outside the mucosa (anterior and posterior to it), and the external sphincter was a less defined hyperechoic area outside the internal sphincter layers (Fig. 1).

The second point was about the application of Doppler technology to HD. Its use could be particularly useful after surgery, in example to screen symptomatic patients for HD recurrence with an easy, quick and non-invasive way. The peculiarity of TPUS, due to the absence of internal compression of the anorectal mucosa, allows the visualization of unaltered vascular flows. Doppler images collected on the three axial planes depicted two patterns, one clearly resembling a pulsatile flow (vascular pattern) and the second consisting of small, scattered dots, without clear pulsations. Our hypothesis was that such patterns corresponded to internal residual — or recurrent — congested vessels [20]. However, it was necessary at this stage to compare US images with a gold-standard method in order to test this hypothesis. We preferred as gold-standard technique the endoscopic retroflexion manoeuvre over anoscopy to minimize, as much as possible, the local vessels compression. Results showed that the “vascular” US pattern had a significant correlation with endoscopic engorged vessels. These were mostly located at the anorectal junction, visible in retroflexion, and presented as one or more close to the MM scars. The statistical analysis showed that the vascular pattern had a positive predictive value of 66.7%, in other words two out of three patients with the vascular pattern have an internal recurrence. Although statistically significant, this predictive value implies that TPUS alone cannot replace endoscopy in identifying all cases of recurrence. Still, it represents a useful, non-invasive first-line screening tool, especially in settings where endoscopy is not immediately available or in patients for whom invasive procedures are poorly tolerated. Even more interesting, the negative predictive value is 100%, in other words all patients with the scattered pattern had no signs of endoscopic pattern of internal recurrence, rendering useful as an initial screening tool in the outpatient clinic. This finding has substantial practical implications: in symptomatic patients with a “scattered” Doppler pattern, clinicians may safely avoid or defer endoscopic evaluation, reserving it only for those with vascular signals. This could help optimize resource use, reduce patient discomfort, and streamline follow-up workflows.

Over the last decades, the US study of pelvic floor diseases progressively gained a widespread acceptance. Clinical indications and applications increased over the years, as well as the number of published studies. Results achieved further reinforced the theory of an integrated approach between the urologist, gynaecologist and colorectal surgeon [1, 2]. Among the various approaches available — TPUS, transvaginal and endoanal — the latter opened the way to more specific and dedicated studies of the anal canal. Its use mainly focused on perianal fistulas (including both idiopathic cryptoglandular and perineal Crohn’s disease), faecal incontinence and staging of anorectal neoplasms [21]. Initial bi-dimensional endoanal probes allowed the anatomical definition of anal sphincters, their relationship with fistulas, and the local level of invasion of neoplasms [21]. The introduction of three-dimensional softwares achieved a better definition of images, further elucidated the regional anatomy and the relationship with the diseases, and expanded indications [21].

Despite the well-known advantages of the endoanal US, it requires a dedicated probe with increased costs, therefore its diffusion is currently limited to tertiary care or specialized colorectal centers making it less accessible in smaller or general healthcare centers. TPUS can be performed with common transabdominal probes generally found in nearly all healthcare settings (including rural clinics and small hospitals), therefore has a more capillary distribution. Furthermore, the less invasive external probes, compared to endoanal ones, could be advantageous to achieve images closer to reality instead of compressing soft vascular tissues and therefore altering the local blood flows. Therefore, Doppler technology could be applied to investigate the true vascular component of diseases, as in example HD [22]. Finally, TPUS overcomes limitations of transvaginal US that can be performed only in women [11].

Significant limitations are present in the current study. The lack of preoperative TPUS assessments of HD patients, as well as a control group of healthy subjects, hinders baseline comparisons, the assessment of confounding factors and a correct analysis of variations induced by surgery. The qualitative classification of patterns observed (vascular vs. scattered) is subjective and should be integrated with more objective quantitative methods (i.e., Doppler measurements of vascular flows). The small sample size of patients that agreed to participate (*n* = 23) out of the 90 available introduces a selection bias; therefore, our results need to be confirmed in larger studies, already underway. Finally, the TPUS methodology, although easy to learn and widely available, still requires a formal training to be properly performed and interpreted.

## Conclusions

Our results have shown that TPUS can be safely applied to the long-term postoperative follow-up of MM and could help screen patients before more invasive investigations are performed, as the 100% negative predictive value of the vascular pattern automatically excludes all patients with endoscopic signs of recurrence when a scattered pattern is present. Future research should involve confirmation of our results in larger cohort studies, quantify the vascular flows through the Doppler measurements, include preoperative flow assessments in order to compare them with postoperative changes, and assess the potential advantages conferred by AI-based image analysis softwares.

## Data Availability

The data that support the findings of this study are not openly available due to reasons of sensitivity and are available from the corresponding author upon reasonable request.

## References

[1] Santoro GA (2017J) Imaging the pelvic floor. Tech Coloproctol 21(7):497–499 28776107 10.1007/s10151-017-1668-y

[2] Santoro GA, Wieczorek AP, Dietz HP, Mellgren A, Sultan AH, Shobeiri SA et al (2011A) State of the art: an integrated approach to pelvic floor ultrasonography. Ultrasound Obstet Gynecol 37(4):381–396 20814874 10.1002/uog.8816

[3] Albuquerque A, Pereira E (2016A 28) Current applications of transperineal ultrasound in gastroenterology. World J Radiol 8(4):370–377 27158423 10.4329/wjr.v8.i4.370 PMC4840194

[4] Plaikner M, Loizides A, Peer S, Aigner F, Pecival D, Zbar A et al (2014F) Transperineal ultrasonography as a complementary diagnostic tool in identifying acute perianal sepsis. Tech Coloproctol 18(2):165–171 23681302 10.1007/s10151-013-1031-x

[5] Lavazza A, Maconi G (2019J) Transperineal ultrasound for assessment of fistulas and abscesses: a pictorial essay. J Ultrasound 22(2):241–249 31066004 10.1007/s40477-019-00381-6 PMC6531524

[6] Shokoohi H, Pyle M, Frasure SE, Dimbil U, Pourmand A (2019N) Point-of-care transperineal ultrasound to diagnose abscess in the emergency department. Clin Pract Cases Emerg Med 3(4):349–353 31763585 10.5811/cpcem.2019.6.43514 PMC6861055

[7] Stewart LK, McGee J, Wilson SR (2001S) Transperineal and transvaginal sonography of perianal inflammatory disease. AJR Am J Roentgenol 177(3):627–632 11517059 10.2214/ajr.177.3.1770627

[8] Wright EK, Novak KL, Lu C, Panaccione R, Ghosh S, Wilson SR. Transperineal ultrasonography in perianal Crohn disease: a valuable imaging modality. Can J Gastroenterol Hepatol. 2015 Nov-Dec;29(8):445–7. 10.1155/2015/120123 PMC4699595 25996615

[9] Jung JH, Ryu YJ, Kim JY, Yang HR (2022O) Transperineal ultrasonography for treatment response evaluation in children with perianal Crohn’s disease. Ultrasonography 41(4):770–781 36059211 10.14366/usg.22057 PMC9532198

[10] Degirmenci Y, Steetskamp J, Schwab R, Hasenburg A, Schepers M, Shehaj I, et al. Functional assessment of anal sphincter with transperineal ultrasound and its relationship to anal continence. Diagnostics (Basel). 2024 Nov 21;14(23). 10.3390/diagnostics14232614 PMC11640527 39682523

[11] Zbar AP, Murison R (2010J) Transperineal ultrasound in the assessment of haemorrhoids and haemorrhoidectomy: a pilot study. Tech Coloproctol 14(2):175–179 20390316 10.1007/s10151-010-0572-5

[12] von Elm E, Altman DG, Egger M, Pocock SJ, GÃ¸tzsche PC, Vandenbroucke JP. The Strengthening the Reporting of Observational Studies in Epidemiology (STROBE) statement: guidelines for reporting observational studies. Lancet. 2007 Oct 20;370(9596):1453–7. 10.1016/S0140-6736(07)61602-X 18064739

[13] Goligher J. Haemorrhoids or piles. In: Goligher J, Duthie H, HH N, eds. Surgery of the anus, rectum and colon. 4th ed. ed. London: Baillière Tindall 1980:96.

[14] Gallo G, Martellucci J, Sturiale A, Clerico G, Milito G, Marino F et al (2020F) Consensus statement of the Italian society of colorectal surgery (SICCR): management and treatment of hemorrhoidal disease. Tech Coloproctol 24(2):145–164 31993837 10.1007/s10151-020-02149-1 PMC7005095

[15] Kuiper SZ, Kimman ML, Van Tol RR, Waardenburg SF, Van Kuijk SMJ, Dirksen CD et al (2022A) Patient reported outcome measure-haemorrhoidal impact and satisfaction score (PROM-HISS): development, reliability and construct validity. Colorectal Dis 24(8):992–999 35119715 10.1111/codi.16079 PMC9544465

[16] Huang WC, Yang SH, Yang JM (2007A) Three-dimensional transperineal sonographic characteristics of the anal sphincter complex in nulliparous women. Ultrasound Obstet Gynecol 30(2):210–220 17659660 10.1002/uog.4083

[17] Peschers UM, DeLancey JO, Schaer GN, Schuessler B (1997S) Exoanal ultrasound of the anal sphincter: normal anatomy and sphincter defects. Br J Obstet Gynaecol 104(9):999–1003 9307524 10.1111/j.1471-0528.1997.tb12056.x

[18] Valsky DV, Messing B, Petkova R, Savchev S, Rosenak D, Hochner-Celnikier D et al (2007F) Postpartum evaluation of the anal sphincter by transperineal three-dimensional ultrasound in primiparous women after vaginal delivery and following surgical repair of third-degree tears by the overlapping technique. Ultrasound Obstet Gynecol 29(2):195–204 17219371 10.1002/uog.3923

[19] GuzmÃ¡n Rojas RA, Shek KL, Langer SM, Dietz HP. Prevalence of anal sphincter injury in primiparous women. Ultrasound Obstet Gynecol. 2013 Oct;42(4):461–6. 10.1002/uog.12481 23576493

[20] Landolfi V, Brusciano L, Gambardella C, Tolone S, Del Genio G, Grossi U et al (2022F) Long-term outcomes of sectorial longitudinal augmented prolapsectomy for asymmetric muco-hemorrhoidal prolapse: an observational study of 433 consecutive patients. Surg Innov 29(1):27–34 33830810 10.1177/15533506211007292

[21] Gravante G, Giordano P (2008J) The role of three-dimensional endoluminal ultrasound imaging in the evaluation of anorectal diseases: a review. Surg Endosc 22(7):1570–1578 18401655 10.1007/s00464-008-9865-4

[22] Tarasconi A, Perrone G, Davies J, Coimbra R, Moore E, Azzaroli F et al (2021S 16) Anorectal emergencies: WSES-AAST guidelines. World J Emerg Surg 16(1):48 34530908 10.1186/s13017-021-00384-x PMC8447593

